# 3-Chloro-*N*′-(4-hy­droxy-3-nitro­benzyl­idene)benzohydrazide methanol disolvate

**DOI:** 10.1107/S1600536811021568

**Published:** 2011-06-11

**Authors:** Zhen Zhang

**Affiliations:** a Experimental Center, Linyi University, Linyi Shandong 276005, People’s Republic of China

## Abstract

In the title compound, C_14_H_10_ClN_3_O_4_·2CH_4_O, the main mol­ecule is in an *E* configuration with respect to the methyl­idene unit. The dihedral angle between the mean planes of the two benzene rings is 1.9 (3)°. In the crystal, inter­molecular N—H⋯O, O—H⋯O and bifurcated O—H⋯(O, O) hydrogen bonds link the components into sheets parallel to (100). An intra­molecular O—H⋯O hydrogen bond is also present.

## Related literature

For the biological applications of hydrazone compounds, see: Ajani *et al.* (2010 ▶); Avaji *et al.* (2009 ▶); Fan *et al.* (2010 ▶); Rasras *et al.* (2010 ▶). For related hydrazone structures, see: Zhang (2011*a* ▶,*b* ▶); Ahmad *et al.* (2010 ▶); Ban (2010 ▶); Ji & Lu (2010 ▶); Shalash *et al.* (2010 ▶).
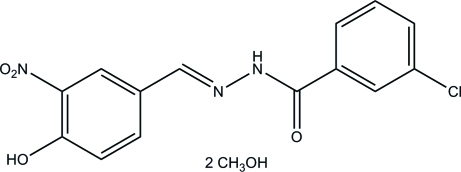


## Experimental

### 

#### Crystal data

C_14_H_10_ClN_3_O_4_·2CH_4_O
*M* *_r_* = 383.78
Monoclinic, 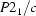

*a* = 7.626 (2) Å
*b* = 18.846 (5) Å
*c* = 12.739 (3) Å
β = 94.689 (4)°
*V* = 1824.6 (8) Å^3^
*Z* = 4
Mo *K*α radiation
μ = 0.25 mm^−1^
*T* = 298 K
0.23 × 0.23 × 0.21 mm

#### Data collection

Bruker SMART APEX CCD diffractometer
Absorption correction: multi-scan (*SADABS*; Bruker, 2009 ▶) *T*_min_ = 0.945, *T*_max_ = 0.950
7726 measured reflections
3695 independent reflections
1842 reflections with *I* > 2σ(*I*)
*R*_int_ = 0.048

#### Refinement

*R*[*F* ^2^ > 2σ(*F* ^2^)] = 0.067
*wR*(*F* ^2^) = 0.208
*S* = 1.06
3695 reflections
243 parameters
1 restraint
H atoms treated by a mixture of independent and constrained refinement
Δρ_max_ = 0.76 e Å^−3^
Δρ_min_ = −0.26 e Å^−3^

### 

Data collection: *APEX2* (Bruker, 2009 ▶); cell refinement: *SAINT* (Bruker, 2009 ▶); data reduction: *SAINT*; program(s) used to solve structure: *SHELXTL* (Sheldrick, 2008 ▶); program(s) used to refine structure: *SHELXTL*; molecular graphics: *SHELXTL*; software used to prepare material for publication: *SHELXTL*.

## Supplementary Material

Crystal structure: contains datablock(s) global, I. DOI: 10.1107/S1600536811021568/lh5265sup1.cif

Structure factors: contains datablock(s) I. DOI: 10.1107/S1600536811021568/lh5265Isup2.hkl

Additional supplementary materials:  crystallographic information; 3D view; checkCIF report

## Figures and Tables

**Table 1:** Hydrogen-bond geometry (Å, °)

*D*—H⋯*A*	*D*—H	H⋯*A*	*D*⋯*A*	*D*—H⋯*A*
O3—H3*A*⋯O1	0.82	1.96	2.618 (4)	137
O3—H3*A*⋯O5	0.82	2.26	2.896 (4)	135
O6—H6⋯O5	0.82	1.88	2.700 (5)	179
N3—H3⋯O6^i^	0.89 (1)	1.99 (2)	2.834 (4)	158 (4)
O5—H5⋯O4^ii^	0.82	1.96	2.779 (4)	173

Symmetry codes: (i) 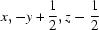; (ii) 
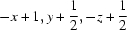
.

**Fig. 1.**
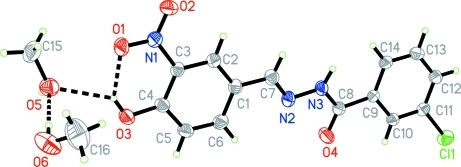
The molecular structure of the title compound, showing displacement ellipsoids at the 30% probability level. Hydrogen bonds are shown as dashed lines.

**Fig. 2.**
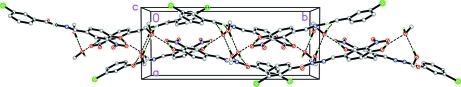
The packing of the title compound. Hydrogen bonds are shown as dashed lines. Only H atoms involved in hydrogen bonds are shown.

## supplementary crystallographic information

### Comment

Hydrazone compounds have received much attention due to their potential applications in biological chemistry (Ajani *et al.*, 2010; Avaji *et al.*, 2009; Fan *et al.*, 2010; Rasras *et al.*, 2010). As a continuation of this work on hydrazone compounds the structure of the title compound is reported herein.

The asymmetric unit of title compound, Fig. 1, contains a hydrazone molecule and two methanol molecules. The hydrazone molecule assumes an *E* configuration with respect to the methylidene unit. The dihedral angle between the mean planes of the two benzene rings is 1.9 (3)°. The bond lengths are comparable to those observed in similar hydrazone compounds (Zhang, 2011*a*,b; Ahmad *et al.*, 2010; Ban, 2010; Ji & Lu, 2010; Shalash *et al.*, 2010). In the crystal, intermolecular N—H···O and O—H···O hydrogen bonds link the components into two dimensional sheets parallel to (100). An intramolecular O-H···O hydrogen bond is also present (Table 1, Fig. 2).

### Experimental

A methanol solution (50 ml) of 3-chlorobenzohydrazide (0.01 mol) and 4-hydroxy-3-nitrobenzaldehyde (0.01 mol) was stirred at room temperature for 30 min to give a yellow solution. Yellow block-shaped single crystals suitable for X-ray diffraction were formed by slow evaporation of the solution in air.

### Refinement

The amino atom, H3, was in from a difference Fourier map and refined isotropically, with the N—H distance restrained to 0.90 (1) Å. The remaining H atoms were positioned geometrically and refined using the riding-model approximation, with C—H = 0.93–0.96 Å, O—H = 0.82 Å, and with *U*_iso_(H) = 1.2*U*_eq_(C) and 1.5*U*_eq_(O).

### Crystal data

**Table:**

C_14_H_10_ClN_3_O_4_·2CH_4_O	*F*(000) = 800
*M**_r_* = 383.78	*D*_x_ = 1.397 Mg m^−^^3^
Monoclinic, *P*2_1_/*c*	Mo *K*α radiation, λ = 0.71073 Å
Hall symbol: -P 2ybc	Cell parameters from 954 reflections
*a* = 7.626 (2) Å	θ = 2.5–24.5°
*b* = 18.846 (5) Å	µ = 0.25 mm^−^^1^
*c* = 12.739 (3) Å	*T* = 298 K
β = 94.689 (4)°	Block, yellow
*V* = 1824.6 (8) Å^3^	0.23 × 0.23 × 0.21 mm
*Z* = 4	

### Data collection

**Table:**

Bruker SMART APEX CCD diffractometer	3695 independent reflections
Radiation source: fine-focus sealed tube	1842 reflections with *I* > 2σ(*I*)
graphite	*R*_int_ = 0.048
ω scans	θ_max_ = 26.5°, θ_min_ = 2.7°
Absorption correction: multi-scan (*SADABS*; Bruker, 2009)	*h* = −9→9
*T*_min_ = 0.945, *T*_max_ = 0.950	*k* = −22→23
7726 measured reflections	*l* = −15→8

### Refinement

**Table:**

Refinement on *F*^2^	Primary atom site location: structure-invariant direct methods
Least-squares matrix: full	Secondary atom site location: difference Fourier map
*R*[*F*^2^ > 2σ(*F*^2^)] = 0.067	Hydrogen site location: inferred from neighbouring sites
*wR*(*F*^2^) = 0.208	H atoms treated by a mixture of independent and constrained refinement
*S* = 1.06	*w* = 1/[σ^2^(*F*_o_^2^) + (0.0891*P*)^2^] where *P* = (*F*_o_^2^ + 2*F*_c_^2^)/3
3695 reflections	(Δ/σ)_max_ = 0.001
243 parameters	Δρ_max_ = 0.76 e Å^−^^3^
1 restraint	Δρ_min_ = −0.26 e Å^−^^3^

### Special details

**Table:**

Geometry. All e.s.d.'s (except the e.s.d. in the dihedral angle between two l.s. planes) are estimated using the full covariance matrix. The cell e.s.d.'s are taken into account individually in the estimation of e.s.d.'s in distances, angles and torsion angles; correlations between e.s.d.'s in cell parameters are only used when they are defined by crystal symmetry. An approximate (isotropic) treatment of cell e.s.d.'s is used for estimating e.s.d.'s involving l.s. planes.
Refinement. Refinement of *F*^2^ against ALL reflections. The weighted *R*-factor *wR* and goodness of fit *S* are based on *F*^2^, conventional *R*-factors *R* are based on *F*, with *F* set to zero for negative *F*^2^. The threshold expression of *F*^2^ > σ(*F*^2^) is used only for calculating *R*-factors(gt) *etc*. and is not relevant to the choice of reflections for refinement. *R*-factors based on *F*^2^ are statistically about twice as large as those based on *F*, and *R*- factors based on ALL data will be even larger.

### Fractional atomic coordinates and isotropic or equivalent isotropic displacement parameters (Å^2^)

**Table:**

	*x*	*y*	*z*	*U*_iso_*/*U*_eq_	
Cl1	1.06078 (17)	−0.35391 (5)	0.03312 (9)	0.0708 (4)	
N1	0.4832 (5)	0.32118 (17)	−0.0141 (3)	0.0546 (9)	
N2	0.7478 (4)	0.02147 (15)	0.0491 (2)	0.0526 (9)	
N3	0.7737 (5)	−0.03895 (16)	−0.0091 (2)	0.0518 (9)	
O1	0.4617 (5)	0.37833 (14)	0.0290 (2)	0.0781 (10)	
O2	0.4421 (4)	0.31188 (15)	−0.1072 (2)	0.0768 (10)	
O3	0.5851 (4)	0.32945 (13)	0.21283 (19)	0.0652 (9)	
H3A	0.5220	0.3575	0.1780	0.098*	
O4	0.8426 (5)	−0.10265 (14)	0.1374 (2)	0.0773 (10)	
O5	0.3664 (4)	0.45501 (16)	0.2154 (2)	0.0744 (9)	
H5	0.3000	0.4414	0.2587	0.112*	
O6	0.6850 (6)	0.5111 (2)	0.2745 (3)	0.1082 (14)	
H6	0.5888	0.4937	0.2563	0.162*	
C1	0.6684 (5)	0.14309 (18)	0.0505 (3)	0.0474 (10)	
C2	0.5915 (5)	0.19991 (18)	−0.0036 (3)	0.0452 (9)	
H2	0.5575	0.1957	−0.0751	0.054*	
C3	0.5644 (5)	0.26316 (18)	0.0480 (3)	0.0437 (9)	
C4	0.6108 (5)	0.27168 (19)	0.1552 (3)	0.0482 (10)	
C5	0.6913 (6)	0.2144 (2)	0.2073 (3)	0.0566 (11)	
H5A	0.7265	0.2186	0.2787	0.068*	
C6	0.7208 (6)	0.1523 (2)	0.1581 (3)	0.0559 (11)	
H6A	0.7765	0.1153	0.1959	0.067*	
C7	0.6967 (5)	0.07624 (19)	−0.0032 (3)	0.0530 (10)	
H7	0.6772	0.0736	−0.0761	0.064*	
C8	0.8224 (5)	−0.09925 (19)	0.0410 (3)	0.0495 (10)	
C9	0.8502 (5)	−0.16287 (17)	−0.0252 (3)	0.0402 (9)	
C10	0.9332 (5)	−0.22067 (18)	0.0246 (3)	0.0459 (9)	
H10	0.9724	−0.2180	0.0956	0.055*	
C11	0.9576 (5)	−0.28200 (18)	−0.0311 (3)	0.0452 (9)	
C12	0.9009 (5)	−0.2878 (2)	−0.1355 (3)	0.0543 (11)	
H12	0.9173	−0.3296	−0.1722	0.065*	
C13	0.8189 (6)	−0.2303 (2)	−0.1848 (3)	0.0564 (11)	
H13	0.7805	−0.2333	−0.2558	0.068*	
C14	0.7929 (5)	−0.16866 (19)	−0.1309 (3)	0.0498 (10)	
H14	0.7365	−0.1305	−0.1656	0.060*	
H3	0.771 (5)	−0.0374 (19)	−0.0792 (9)	0.060*	
C15	0.2719 (8)	0.4968 (2)	0.1385 (4)	0.0930 (17)	
H15A	0.2107	0.5338	0.1724	0.139*	
H15B	0.1885	0.4676	0.0979	0.139*	
H15C	0.3520	0.5175	0.0929	0.139*	
C16	0.7983 (9)	0.5025 (3)	0.1930 (5)	0.132 (2)	
H16A	0.7729	0.5382	0.1402	0.198*	
H16B	0.7808	0.4563	0.1620	0.198*	
H16C	0.9183	0.5071	0.2215	0.198*	

### Atomic displacement parameters (Å^2^)

**Table:**

	*U*^11^	*U*^22^	*U*^33^	*U*^12^	*U*^13^	*U*^23^
Cl1	0.0871 (10)	0.0429 (6)	0.0794 (8)	0.0123 (6)	−0.0122 (6)	0.0061 (5)
N1	0.064 (2)	0.048 (2)	0.051 (2)	0.0092 (17)	0.0017 (17)	0.0028 (16)
N2	0.058 (2)	0.0397 (17)	0.0606 (19)	0.0051 (16)	0.0061 (16)	−0.0027 (15)
N3	0.064 (2)	0.0435 (18)	0.0480 (17)	0.0113 (16)	0.0056 (17)	−0.0019 (15)
O1	0.124 (3)	0.0458 (16)	0.0628 (18)	0.0307 (18)	−0.0022 (18)	−0.0022 (14)
O2	0.104 (3)	0.070 (2)	0.0520 (18)	0.0192 (18)	−0.0196 (17)	−0.0038 (14)
O3	0.095 (3)	0.0486 (16)	0.0492 (15)	0.0114 (16)	−0.0097 (15)	−0.0068 (13)
O4	0.127 (3)	0.0578 (17)	0.0454 (16)	0.0261 (18)	−0.0019 (17)	−0.0041 (13)
O5	0.086 (3)	0.077 (2)	0.0608 (19)	0.0012 (19)	0.0064 (17)	0.0053 (15)
O6	0.140 (4)	0.105 (3)	0.078 (2)	−0.042 (3)	0.000 (2)	−0.014 (2)
C1	0.051 (3)	0.039 (2)	0.052 (2)	0.0008 (19)	0.0063 (19)	0.0016 (17)
C2	0.045 (2)	0.044 (2)	0.047 (2)	−0.0023 (18)	0.0038 (17)	−0.0009 (16)
C3	0.049 (3)	0.0349 (19)	0.047 (2)	0.0026 (18)	0.0043 (18)	0.0056 (15)
C4	0.057 (3)	0.042 (2)	0.044 (2)	−0.0030 (19)	−0.0013 (19)	−0.0009 (17)
C5	0.070 (3)	0.054 (2)	0.043 (2)	0.003 (2)	−0.007 (2)	0.0006 (18)
C6	0.064 (3)	0.047 (2)	0.055 (2)	0.006 (2)	−0.002 (2)	0.0099 (18)
C7	0.060 (3)	0.045 (2)	0.054 (2)	0.001 (2)	0.007 (2)	−0.0011 (18)
C8	0.057 (3)	0.042 (2)	0.049 (2)	0.0069 (19)	0.0000 (19)	−0.0014 (17)
C9	0.039 (2)	0.040 (2)	0.0413 (19)	0.0031 (17)	0.0015 (16)	−0.0003 (15)
C10	0.049 (3)	0.044 (2)	0.044 (2)	0.0033 (19)	0.0000 (18)	−0.0002 (16)
C11	0.044 (2)	0.0346 (19)	0.057 (2)	−0.0009 (17)	0.0000 (18)	0.0045 (16)
C12	0.059 (3)	0.047 (2)	0.056 (2)	0.003 (2)	−0.003 (2)	−0.0118 (18)
C13	0.062 (3)	0.059 (3)	0.047 (2)	0.004 (2)	−0.006 (2)	−0.0042 (19)
C14	0.051 (3)	0.047 (2)	0.050 (2)	0.0079 (19)	−0.0027 (19)	0.0044 (17)
C15	0.113 (5)	0.074 (3)	0.092 (3)	0.034 (3)	0.012 (3)	0.018 (3)
C16	0.136 (6)	0.136 (6)	0.133 (5)	−0.021 (5)	0.061 (5)	−0.028 (5)

### Geometric parameters (Å, °)

**Table:**

Cl1—C11	1.738 (3)	C5—C6	1.355 (5)
N1—O2	1.214 (4)	C5—H5A	0.9300
N1—O1	1.226 (4)	C6—H6A	0.9300
N1—C3	1.458 (4)	C7—H7	0.9300
N2—C7	1.272 (4)	C8—C9	1.491 (5)
N2—N3	1.382 (4)	C9—C14	1.386 (5)
N3—C8	1.341 (4)	C9—C10	1.387 (4)
N3—H3	0.893 (10)	C10—C11	1.377 (5)
O3—C4	1.336 (4)	C10—H10	0.9300
O3—H3A	0.8200	C11—C12	1.369 (5)
O4—C8	1.227 (4)	C12—C13	1.377 (5)
O5—C15	1.409 (5)	C12—H12	0.9300
O5—H5	0.8200	C13—C14	1.371 (5)
O6—C16	1.413 (6)	C13—H13	0.9300
O6—H6	0.8200	C14—H14	0.9300
C1—C2	1.378 (5)	C15—H15A	0.9600
C1—C6	1.406 (5)	C15—H15B	0.9600
C1—C7	1.458 (5)	C15—H15C	0.9600
C2—C3	1.385 (5)	C16—H16A	0.9600
C2—H2	0.9300	C16—H16B	0.9600
C3—C4	1.392 (5)	C16—H16C	0.9600
C4—C5	1.385 (5)		
			
O2—N1—O1	122.1 (3)	O4—C8—C9	120.8 (3)
O2—N1—C3	119.1 (3)	N3—C8—C9	117.4 (3)
O1—N1—C3	118.8 (3)	C14—C9—C10	118.6 (3)
C7—N2—N3	116.0 (3)	C14—C9—C8	124.4 (3)
C8—N3—N2	119.3 (3)	C10—C9—C8	117.0 (3)
C8—N3—H3	119 (2)	C11—C10—C9	119.9 (3)
N2—N3—H3	121 (2)	C11—C10—H10	120.0
C4—O3—H3A	109.5	C9—C10—H10	120.0
C15—O5—H5	109.5	C12—C11—C10	121.5 (3)
C16—O6—H6	109.5	C12—C11—Cl1	119.3 (3)
C2—C1—C6	117.8 (3)	C10—C11—Cl1	119.1 (3)
C2—C1—C7	120.7 (3)	C11—C12—C13	118.4 (3)
C6—C1—C7	121.5 (3)	C11—C12—H12	120.8
C1—C2—C3	120.4 (3)	C13—C12—H12	120.8
C1—C2—H2	119.8	C14—C13—C12	121.2 (4)
C3—C2—H2	119.8	C14—C13—H13	119.4
C2—C3—C4	122.0 (3)	C12—C13—H13	119.4
C2—C3—N1	117.5 (3)	C13—C14—C9	120.4 (3)
C4—C3—N1	120.6 (3)	C13—C14—H14	119.8
O3—C4—C5	116.8 (3)	C9—C14—H14	119.8
O3—C4—C3	126.6 (3)	O5—C15—H15A	109.5
C5—C4—C3	116.6 (3)	O5—C15—H15B	109.5
C6—C5—C4	122.4 (4)	H15A—C15—H15B	109.5
C6—C5—H5A	118.8	O5—C15—H15C	109.5
C4—C5—H5A	118.8	H15A—C15—H15C	109.5
C5—C6—C1	120.9 (3)	H15B—C15—H15C	109.5
C5—C6—H6A	119.6	O6—C16—H16A	109.5
C1—C6—H6A	119.6	O6—C16—H16B	109.5
N2—C7—C1	120.4 (3)	H16A—C16—H16B	109.5
N2—C7—H7	119.8	O6—C16—H16C	109.5
C1—C7—H7	119.8	H16A—C16—H16C	109.5
O4—C8—N3	121.8 (3)	H16B—C16—H16C	109.5

### Hydrogen-bond geometry (Å, °)

**Table:**

*D*—H···*A*	*D*—H	H···*A*	*D*···*A*	*D*—H···*A*
O3—H3A···O1	0.82	1.96	2.618 (4)	137
O3—H3A···O5	0.82	2.26	2.896 (4)	135
O6—H6···O5	0.82	1.88	2.700 (5)	179
N3—H3···O6^i^	0.89 (1)	1.99 (2)	2.834 (4)	158 (4)
O5—H5···O4^ii^	0.82	1.96	2.779 (4)	173

Symmetry codes: (i) *x*, −*y*+1/2, *z*−1/2; (ii) −*x*+1, *y*+1/2, −*z*+1/2.
